# Highly Multiplexed Phenotyping of Immunoregulatory Proteins in the Tumor Microenvironment by CODEX Tissue Imaging

**DOI:** 10.3389/fimmu.2021.687673

**Published:** 2021-05-19

**Authors:** Darci Phillips, Christian M. Schürch, Michael S. Khodadoust, Youn H. Kim, Garry P. Nolan, Sizun Jiang

**Affiliations:** ^1^ Department of Pathology, Stanford University School of Medicine, Stanford, CA, United States; ^2^ Department of Dermatology, Stanford University School of Medicine, Stanford, CA, United States; ^3^ Department of Pathology and Neuropathology, University Hospital and Comprehensive Cancer Center Tübingen, Tübingen, Germany; ^4^ Division of Oncology, Stanford University School of Medicine, Stanford, CA, United States

**Keywords:** immunotherapy, CODEX multiplexed tissue imaging, immunophenotyping, immunoregulatory proteins, tumor microenvironment, cancer immunity, lymphoma

## Abstract

Immunotherapies are revolutionizing cancer treatment by boosting the natural ability of the immune system. In addition to antibodies against traditional checkpoint molecules or their ligands (i.e., CTLA-4, PD-1, and PD-L1), therapies targeting molecules such as ICOS, IDO-1, LAG-3, OX40, TIM-3, and VISTA are currently in clinical trials. To better inform clinical care and the design of therapeutic combination strategies, the co-expression of immunoregulatory proteins on individual immune cells within the tumor microenvironment must be robustly characterized. Highly multiplexed tissue imaging platforms, such as CO-Detection by indEXing (CODEX), are primed to meet this need by enabling >50 markers to be simultaneously analyzed in single-cells on formalin-fixed paraffin-embedded (FFPE) tissue sections. Assembly and validation of antibody panels is particularly challenging, with respect to the specificity of antigen detection and robustness of signal over background. Herein, we report the design, development, optimization, and application of a 56-marker CODEX antibody panel to eight cutaneous T cell lymphoma (CTCL) patient samples. This panel is comprised of structural, tumor, and immune cell markers, including eight immunoregulatory proteins that are approved or currently undergoing clinical trials as immunotherapy targets. Here we provide a resource to enable extensive high-dimensional, spatially resolved characterization of the tissue microenvironment across tumor types and imaging modalities. This framework provides researchers with a readily applicable blueprint to study tumor immunology, tissue architecture, and enable mechanistic insights into immunotherapeutic targets.

## Introduction

Immunotherapies work through the blockage or stimulation of immunoregulatory proteins to enhance the body’s innate ability to target and destroy tumor cells. CTLA-4, PD-1, and PD-L1 are the most widely studied inhibitory checkpoint molecules, and drugs targeting these proteins have revolutionized treatment for numerous solid and hematological malignancies (1). Unfortunately, only about 20% of patients derive long-lasting benefits from current immunotherapies (2). Novel therapies targeting inhibitory (e.g., IDO-1, LAG-3, TIGIT, TIM-3, VISTA) and stimulatory (e.g., ICOS, GITR, OX40, 4-IBB) proteins within the tumor microenvironment (TME) are under active investigation (3–7). Clinical trials are also underway to combine these novel pre-clinical treatments with established anti-CTLA-4 (i.e., ipilimumab) and anti-PD-1 (i.e., pembrolizumab and nivolumab) therapies for greater effect (7–13). This expanding list of targets underscores the urgent need to characterize the expression of immunoregulatory proteins, in the native context of individual cells within the TME, to drive immunotherapy selection for cancer patients (14).

Clinical staining of immunoregulatory proteins is routinely performed with conventional one- or two-color immunohistochemistry (IHC). Studying more than two markers either requires a careful selection of primary antibodies (i.e., raised in different species to prevent cross-reactivity with the secondary antibodies) or the use of consecutive tissue sections, which is problematic for studying samples with low tissue availability and makes it extremely difficult to co-localize markers at the single-cell level. Therefore, conventional IHC drastically limits accurate classification of both cell-type and function (e.g., reactive PD-1^+^ CD4^+^ T cells versus immunosuppressive PD-1^+^ FOXP3^+^ CD4^+^ T cells). This in turn limits a comprehensive understanding of the TME and the factors underlying immunotherapy responsiveness.

The emergence of multiplexed tissue imaging has enabled researchers to overcome these challenges and to further our understanding of cancer immunotherapy. Recent work exemplifies how multiplexed IHC (mIHC) is superior to single-plex PD-L1 IHC, tumor mutational burden, or gene expression profiling for predicting response to PD-1/PD-L1 blockade therapies across 10 different solid tumors (15). CO-Detection by indEXing (CODEX), a commercialized and accessible multiplexed tissue imaging platform (Akoya Biosciences, Menlo Park, California, USA), uses oligonucleotide-conjugated antibodies and sequential fluorescent reporters, to detect up to 60 markers simultaneously in a single tissue section at resolutions that resolve individual cells. As such, CODEX generates detailed information on the distribution of different cellular phenotypes, while maintaining the morphological context of healthy and diseased tissues (16). Since the CODEX method was first described in 2018 (17), this technology has been successfully adapted for use in FFPE tissues (16, 18) and applied to immunophenotype solid (16) and hematological (19) malignancies. Establishment of a companion computational framework has been crucial for processing raw CODEX imaging data, mapping cellular interactions, and analyzing cellular neighborhoods (16–21). These studies have accelerated the discovery of new immune cell subsets (16, 17) and biomarkers (19), and correlated spatial organization with cancer prognosis (16) and immunotherapeutic outcomes (19).

A fundamental aspect of the CODEX methodology relies upon a well-designed and validated antibody panel. Combining up to 60 markers in a single experiment requires that all antibodies to stain robustly under the same antigen retrieval condition, and that antibody performance is optimized by the imaging order. Herein, we describe the establishment of a 56-marker panel to analyze FFPE cutaneous T cell lymphoma (CTCL) tissues by CODEX. This panel comprises immune, tumor, and structural (e.g., epithelial, stromal, vascular) markers. It also includes eight immunoregulatory proteins—ICOS, IDO-1, LAG-3, PD-1, PD-L1, OX40, Tim-3, and VISTA—to simultaneously phenotype, localize, and quantify these functional molecules on individual cells within the TME, adding important insights to the field of cancer immunotherapy. This work serves as a blueprint for customizing CODEX antibody panels and provides researchers and clinicians with a working antibody panel for high-dimensional characterization of the TME, with broad adaptability to different malignancies of interest or alternative imaging platforms, such as Imaging Mass Cytometry (IMC) (22–24), Multiplexed Ion Beam Imaging (MIBI) (25–28), and tissue-based cyclic immunofluorescence (t-CyCIF) (29).

## Methods

### Tissue Material

Skin tumor samples were obtained from CTCL patients treated at Stanford University. Written informed consent was obtained from all patients. The use of their tissues for this research was fully anonymized and approved by the Stanford University IRB Administrative Panels on Human Subjects in Medical Research (HSR 46894). FFPE histology blocks were generated according to standard pathology procedures (30). A tissue microarray was then created from eight CTCL specimens (**Supplementary Table 1**). Tissue microarray cores were 0.6 mm in diameter and acquired from the most heavily tumor-infiltrated area of the biopsy. The tissue microarray was sectioned at 4-µm thickness and mounted onto Vectabond™-treated (Vector Laboratories, Burlingame, CA, USA; #SP-1800) square glass coverslips (Electron Microscopy Sciences, Hatfield, PA, USA; #72204-01), as previously described (16, 19).

### Antibodies and DNA Oligonucleotide Conjugation

Commercially available purified, carrier-free monoclonal and polyclonal anti-human antibodies (**Table 1**) were conjugated to maleimide-modified short DNA oligonucleotides [TriLink Biotechnologies, San Diego, CA, USA; for detailed oligonucleotide sequences see (16)] at a 2:1 weight/weight ratio of oligonucleotide to antibody, as previously described (16, 18, 19). Conjugated antibodies were subsequently stored at 4°C, where they remained stable for at least 1 year. Conjugated antibodies were titrated and validated under the supervision of a board-certified pathologist (C.M.S.) and confirmed with an online database (The Human Protein Atlas; www.proteinatlas.org) (31).

**Table 1:** Marker panel (56 antibodies and 2 nuclear stains) for CODEX.

Target	Clone	Supplier	Oligo*	Fluorophore	Dilution	Exposure time	Cycle	Channel
CD1a	O10+CA1/71	Novus Biologicals	43	Cy5	1:100	1/2s	19	4
CD2	RPA-2.10	Biolegend	25	Cy5	1:25	1/2s	7	4
CD3	MRQ-39	Cell Marque	77	Cy5	1:100	1/2s	16	4
CD4	EPR6855	Abcam	20	ATTO550	1:100	1/2s	7	3
CD5	UCHT2	Biolegend	75	ATTO550	1:50	1/2s	8	3
CD7	MRQ-56	Cell Marque	63	ATTO550	1:100	1/2s	19	3
CD8	C8/144B	Novus Biologicals	8	Cy5	1:50	1/5s	17	4
CD11b	EPR1344	Abcam	28	Cy5	1:50	1/2s	21	4
CD11c	EP1347Y	Abcam	49	ATTO550	1:50	1/2s	11	3
CD15	MMA	BD Biosciences	14	Alexa488	1:200	1/8.5s	13	2
CD16	D1N9L	Cell Signaling Technology	26	ATTO550	1:100	1/2s	12	3
CD20	rIGEL/773	Novus Biologicals	48	ATTO550	1:200	1/4s	10	3
CD25	4C9	Cell Marque	24	ATTO550	1:100	1/2s	9	3
CD30	BerH2	Cell Marque	57	ATTO550	1:25	1/2s	5	3
CD31	C31.3+C31.7+C31.10	Novus Biologicals	68	ATTO550	1:200	1/5s	24	3
CD34	QBEnd/10	Novus Biologicals	38	ATTO550	1:100	1/4s	22	3
CD38	EPR4106	Abcam	66	ATTO550	1:100	1/5s	23	3
CD45	B11+PD7/26	Novus Biologicals	56	ATTO550	1:400	1/8.5s	26	3
CD45RA	HI100	Biolegend	72	Cy5	1:50	1/2s	15	4
CD45RO	UCH-L1	Biolegend	2	ATTO550	1:100	1/4s	20	3
CD56	MRQ-42	Cell Marque	29	Cy5	1:50	1/2s	3	4
CD57	HCD57	Biolegend	30	ATTO550	1:200	1/4s	16	3
CD68	KP-1	Biolegend	70	Cy5	1:100	1/4s	23	4
CD69	polyclonal	R&D Systems	36	ATTO550	1:200	1/2s	15	3
CD138	B-A38	Novus Biologicals	76	ATTO550	1:100	1/8.5s	25	3
CD162	HECA-452	Novus Biologicals	46	Cy5	1:200	1/8.5s	27	4
CD163	EDHu-1	Novus Biologicals	45	Cy5	1:200	1/4s	21	3
CD194	L291H4	Biolegend	71	Cy5	1:100	1/2s	20	4
CD206	polyclonal	R&D Systems	55	ATTO550	1:100	1/2s	14	3
α-SMA	polyclonal	Abcam	69	Alexa488	1:200	1/3s	10	2
β-catenin	14	BD Biosciences	51	Cy5	1:50	1/2s	18	4
BCL-2	124	Novus Biologicals	41	ATTO550	1:50	1/2s	18	3
CCR6	polyclonal	Novus Biologicals	53	Cy5	1:25	1/2s	5	4
Collagen IV	polyclonal	Abcam	33	Cy5	1:200	1/4s	24	4
Cytokeratin	C11	Biolegend	62	Cy5	1:200	1/5s	22	4
DRAQ5	N/A	Cell Signaling Technology	N/A	Cy5	1:100	1/5s	28	4
EGFR	D38B1	Cell Signaling Technology	58	ATTO550	1:25	1/2s	13	3
FoxP3	236A/E7	Abcam	61	ATTO550	1:100	1/4s	3	3
GATA3	L50-823	Cell Marque	60	Cy5	1:100	1/2s	2	4
Granzyme B	EPR20129-217	Abcam	81	Alexa488	1:200	1/8.5s	14	2
HLA-DR	EPR3692	Abcam	65	ATTO550	1:200	1/4s	17	3
Hoechst 33342	N/A	Thermo Fisher Scientific	N/A	Hoechst	1:1000	1/150s	1-28	1
ICOS	D1K2T	Cell Signaling Technology	74	Cy5	1:100	1/2s	14	4
IDO-1	D5J4E	Cell Signaling Technology	59	Cy5	1:25	1/2s	12	4
Ki-67	B56	BD Biosciences	6	Cy5	1:100	1/5s	8	4
LAG-3	D2G4O	Cell Signaling Technology	42	Cy5	1:25	1/2s	10	4
Mast cell tryptase	AA1	Abcam	44	ATTO550	1:200	1/100s	27	3
MMP-9	L51/82	Biolegend	80	Cy5	1:200	1/6s	26	4
MUC-1	955	Novus Biologicals	15	Alexa488	1:100	1/2s	11	2
OX40	polyclonal	R&D Systems	67	Cy5	1:100	1/2s	9	4
PD-1	D4W2J	Cell Signaling Technology	23	Cy5	1:50	1/2s	11	4
PD-L1	E1L3N	Cell Signaling Technology	11	ATTO550	1:50	1/2s	6	3
Podoplanin	D2-40	Biolegend	32	Cy5	1:200	1/3s	25	4
T-bet	D6N8B	Cell Signaling Technology	5	ATTO550	1:100	1/2s	2	3
TCR-γ/δ	H-41	Santa Cruz Biotechnology	52	ATTO550	1:100	1/2s	4	3
Tim-3	polyclonal	R&D Systems	21	Cy5	1:50	1/2s	4	4
Vimentin	RV202	BD Biosciences	7	Alexa488	1:200	1/4s	12	2
VISTA	D1L2G	Cell Signaling Technology	79	Cy5	1:50	1/2s	13	4

*DNA oligonucleotide sequences are detailed in Table S1C (Schürch, C.M., et al. Cell. 2020; https://doi.org/10.1016/j.cell.2020.07.005) (16).

### CODEX Multiplexed Tissue Staining and Image Acquisition

CODEX staining and imaging were performed as previously described (16, 18, 19). Briefly, the coverslip containing the tissue section was baked at 70°C for 1 hour, deparaffinized in xylene, rehydrated in ethanol, and washed in ddH_2_O before performing heat-induced epitope retrieval with Dako target antigen retrieval solution, pH 9 (Agilent Technologies, Santa Clara, CA, USA; #S236784-2) at 97°C for 10 min on a LabVison PT Module (Thermo Fisher Scientific, #A80400012). The coverslip was subsequently blocked using blocking buffer [S2 buffer containing B1 (1:20), B2 (1:20), B3 (1:20), and BC4 (1:15)] and stained with the 56-marker antibody panel (**Table 1**) to a volume of 100 µl overnight at 4°C. After fixation with 1.6% paraformaldehyde, 100% methanol, and BS3 (Thermo Fisher Scientific, Waltham, MA, USA; #21580), the coverslip was mounted onto a custom-made acrylic plate (Bayview Plastic Solutions, Fremont, CA, USA). Imaging of the CODEX multicycle experiment was performed using an inverted fluorescence microscope (Keyence, Osaka, Japan; model BZ-X710) equipped with a CFI Plan Apo λ 20x/0.75 objective (Nikon, Tokyo, Japan), a microfluidics instrument (Akoya Biosciences, Menlo Park, CA, USA), and CODEX driver software (Akoya Biosciences, Menlo Park, CA, USA). Light exposure times and the order of markers per cycle are outlined in **Table 1**. The Hoechst nuclear stain (Thermo Fisher Scientific; #62249) was acquired in each cycle and DRAQ5 nuclear stain (Cell Signaling Technology, Danvers, MA, USA; #4084L) was acquired in the final cycle. After completion of the multi-cycle reaction, manual hematoxylin and eosin (H&E) staining was performed according to standard pathology procedures (30), and the tissue microarray was re-imaged in brightfield mode.

### Processing and Analysis of CODEX Data

Raw TIFF image files were processed using the CODEX Toolkit, as previously described (18, 19). Briefly, after the data was uploaded, cell segmentation was performed using DRAQ5 nuclear stain. Antibody expression was quantified at the single-cell level and this data was cleaned by gating using CellEngine (https://cellengine.com). This yielded a total of 25,456 cells across the eight tissue microarray cores. The resultant FCS files were imported into the VorteX clustering software (20) and subjected to unsupervised X-shift clustering, with the following 30 parameters: CD1a, CD3, CD4, CD7, CD8, CD11c, CD15, CD20, CD25, CD30, CD31, CD34, CD38, CD45, CD56, CD68, CD138, CD163, CD206, α-smooth muscle actin, cell size, cytokeratin, FoxP3, HLA-DR, Ki-67, mast cell tryptase, PD-1, PD-L1, podoplanin, and vimentin. Cell morphology and size were used to further refine clusters manually and those with similar features were merged, resulting in 18 cell-types. As all specimens did not contain epithelium, and in turn Langerhans cells, clusters with CD11c^+^ dendritic cells and Langerhans cells were merged and classified as antigen presenting cells (APC). This led to 17 cell-types (**Figure 2A**). The expression frequencies of ICOS, IDO-1, LAG-3, PD-1, PD-L1, OX40, Tim-3, and VISTA were determined for CD4^+^ T cells, CD8^+^ T cells, Tregs, M1 macrophages, M2 macrophages, and tumor cells by manual gating in CellEngine (https://cellengine.com).

### Visualizing CODEX Data

Seven-color overlay images with select markers were created in ImageJ (https://imagej.net/). Simulated brightfield IHC images were generated from CODEX fluorescence data using an ImageJ macro (https://bitbucket.org/davemason/makehdab/src/master/). Voronoi diagrams of assigned cell-types were created in Python, with slight modifications to previously described scripts (https://github.com/nolanlab/NeighborhoodCoordination/) (16).

## Results

Here, we describe the development, optimization, and application of a 56-marker antibody panel to characterize the composition, spatial organization, and functional immune status of the TME in FFPE tumors by CODEX (**Table 1**). Marker selection was based on 1) in-house testing of antibody performance in IHC and FFPE-CODEX (16, 18, 19), 2) IHC experience in clinical pathology laboratories, 3) commercially available fluorophore-conjugated antibodies that either in their conjugated or unconjugated forms work in IHC, 4) IHC and FFPE multiplexed tissue imaging publications, and 5) online databases like The Human Protein Atlas (www.proteinatlas.org) (31). Notably, 51 of the 56 antibodies in this panel have been used in other multiplexed imaging studies (**Supplementary Table 2**) (16, 18, 23, 24, 26–29). These 51 antibodies were studied in different tissues (e.g., tonsil, breast cancer, colon cancer, pancreatic cancer, squamous cell carcinoma, etc.), using different modalities (i.e., CODEX, MIBI, IMC, t-CyCIF), and by different research groups (i.e., in different countries, by different operators), which emphasizes the reproducibility of these markers and satisfies an orthogonal strategy for antibody validation (32). The key contribution of the current study is therefore the presentation of a robust, working 56-marker panel focused on immunophenotyping and immunoregulation. Interested researchers can easily purchase, conjugate, and titrate the antibodies shown in **Table 1** as a starting point for their own multiplexed tissue imaging panels.

All antibodies were conjugated to unique maleimide-modified DNA oligonucleotides (with lengths between 10-19 nucleotides), added to tissues that were subjected to high pH (pH 9) antigen retrieval buffer, incubated overnight at 4°C, and tested/titrated in CODEX single-staining experiments. Antibody testing was performed using tonsil (**Supplementary Figure 1**) and a tissue microarray with 16 healthy and 54 cancerous tissues (16, 18) to ensure the inclusion of appropriate positive and negative controls. Conjugated antibodies were stained at a dilution between 1:25 and 1:200 and visually evaluated for subcellular localization (e.g., nucleus, cytoplasm, membrane), cell-type (e.g., immune, tumor, or stromal cells), signal intensity, dynamic range, and signal-to-noise ratio.

After determining the optimal conditions for each conjugated antibody by CODEX single-staining, a 56-marker multicycle experiment was performed on a CTCL tissue microarray compiled from eight patients (**Supplementary Figures 2** and **3A, B**). All antibodies performed as expected when imaged during the CODEX multicycle experiment (**Supplementary Figure 4**), including the seven polyclonal antibodies used in this panel, which showed consistent staining intensity, appropriate spatial expression patterns, and no anomalous cross-reactivity. Importantly, the reproducibility of antibody staining is high within a single CODEX multicycle experiment for a given tissue region (16, 18, 21), between patient samples (**Supplementary Figure 5**) as well as across experiments processed in parallel (19). For all CODEX experiments, the first cycle is a “blank” cycle (i.e., no fluorescent oligonucleotides are added). This is critical for assigning cell-types from imaging data, which consists of subtracting background fluorescence signals that arise from auto-fluorescence and non-specific antibody binding (measured from the “blank” cycle), segmenting the image to identify individual cells, and integrating the staining intensity of all markers on each cell to identify cell-types.

**Figure 1:**
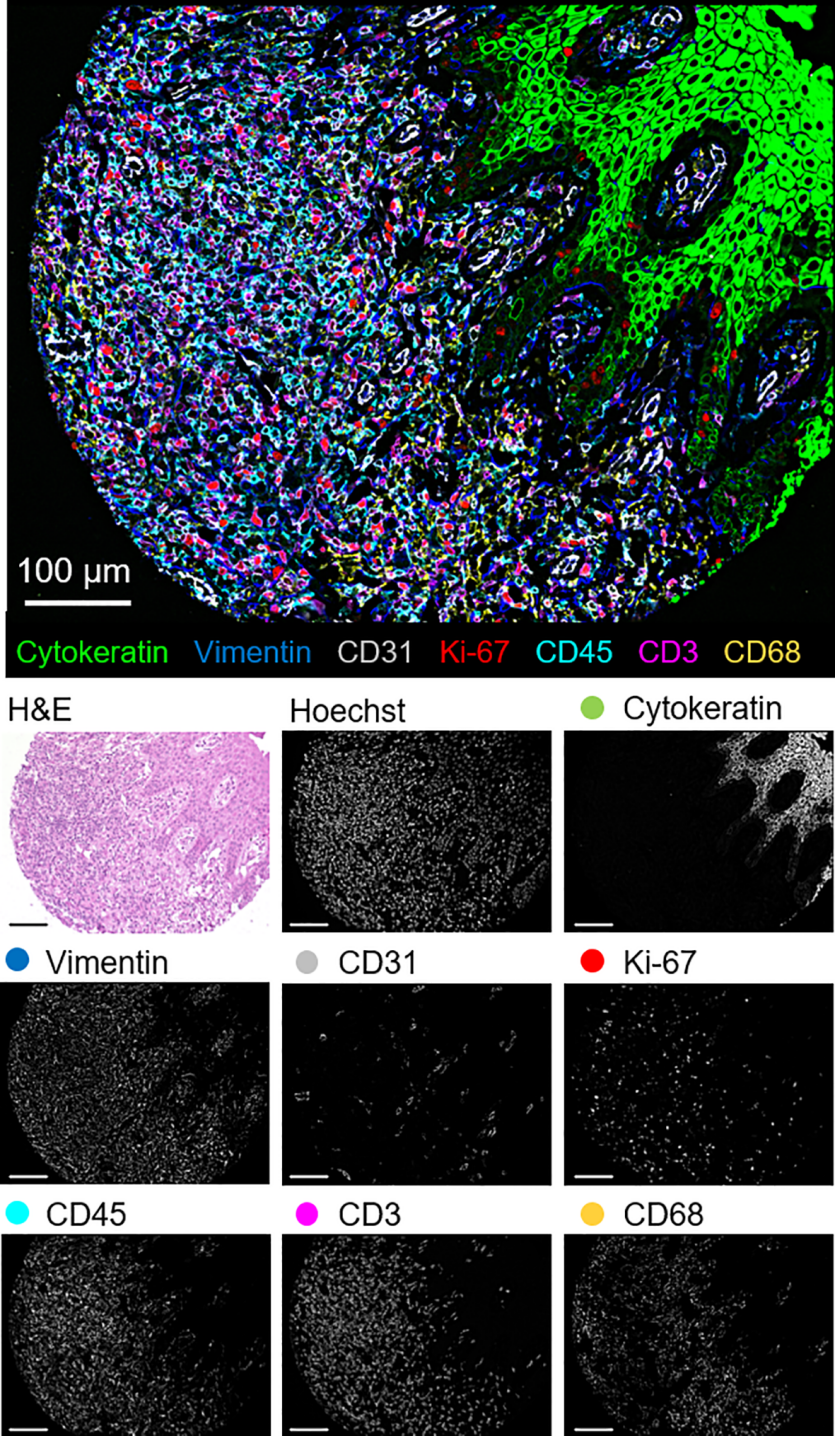
Detection of structural, lymphoid, myeloid, and tumor markers by CODEX in a single CTCL tissue microarray core (patient 6). Seven-color overlay image with cytokeratin (epithelium; green), vimentin (stroma; blue), CD31 (vasculature; gray), Ki-67 (proliferation; red), CD45 (leukocytes; cyan), CD3 (T cells; magenta), and CD68 (macrophages; yellow). Below panels show H&E, Hoechst (nuclear), and individual marker stainings. Scale bars, 100 µm.

The 56-marker panel consists of structural, tumor, and immune markers as well as antibodies against proteins that reflect functional cellular states and immune regulation. A representative seven-color overlay image shows structural, lymphoid, myeloid, and tumor cell markers in a CTCL tissue microarray core (**Figure 1**). Seven-color overlay images for all tissue microarray cores are shown in **Supplementary Figure 3C**.

**Figure 2:**
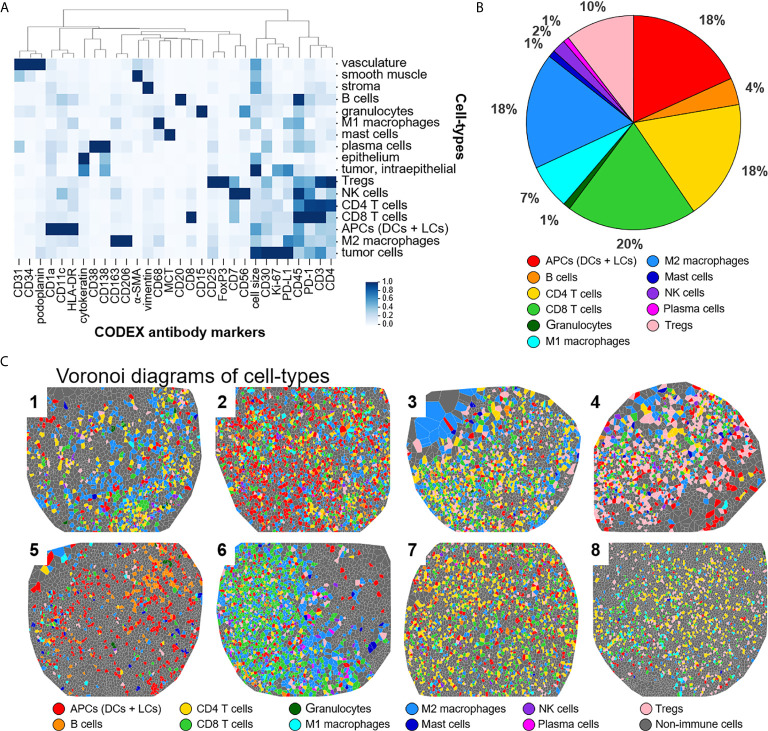
Detecting cell-types by CODEX. **(A)** Heatmap of CODEX-identified cell-types clusters by protein expression; antigen presenting cells (APCs) include dendritic cells (DCs) and Langerhans cells (LCs). **(B)** Immune cell composition in all patients. **(C)** Voronoi diagrams mapping cell-type positions, colored according to the legend.

This panel enables extensive immunophenotyping: 33 of the 56 antibodies recognize antigens specific for T cells, B cells, plasma cells, NK cells, macrophages, dendritic cells, Langerhans cells, granulocytes, and mast cells. In fact, 11 of the 17 identified cell-types were immune specific, where dendritic cells and Langerhans cells were merged and classified as APCs (**Figure 2A**). The immune cell-types include APCs, B cells, CD4^+^ T cells, CD8^+^ T cells, granulocytes, M1 macrophages, M2 macrophages, mast cells, NK cells, plasma cells, and Tregs. The frequency of immune cell subsets across all patients identified populations with high (APCs, 18%; CD4^+^ T cells, 18%; CD8^+^ T cells, 20%; M2 macrophages, 18%; Tregs, 10%), medium (B cells, 4%; M1 macrophages, 7%), and low abundance (NK cells, 2%; granulocytes, 1%; mast cells, 1%; plasma cells, 1%) (**Figure 2B**). Voronoi diagrams (i.e., cell position plots) were used to generate a map of immune cell-type positions (**Figure 2C**). These maps reveal substantial spatial heterogeneity between patients, suggesting dynamic and variable effects of immune surveillance within the TME.

**Figure 3:**
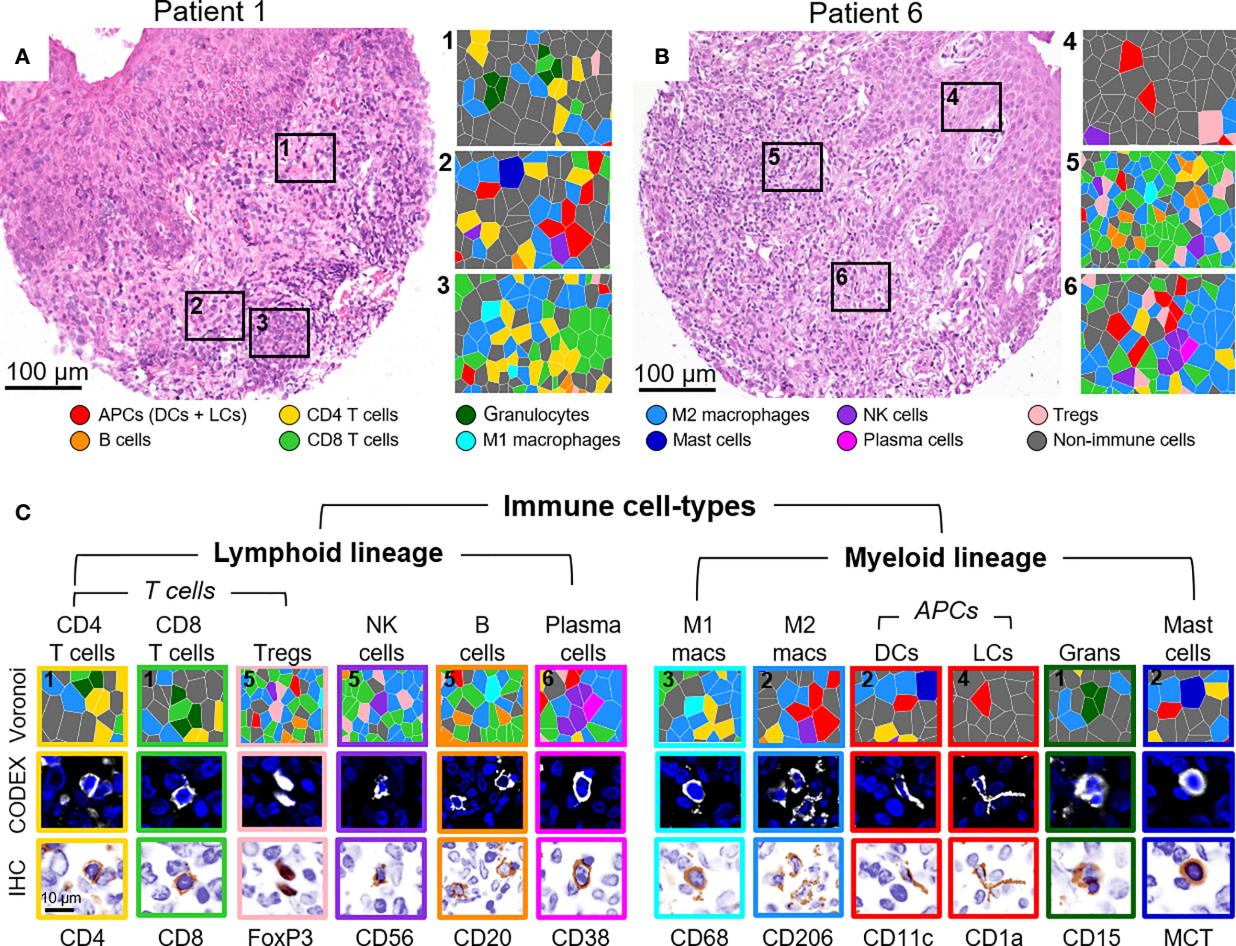
Visualizing immune cell-types by CODEX. **(A, B)** H&E stainings for two different CTCL patients and corresponding Voronoi diagrams from 6 select regions; see **Figure 2B** for full Voronoi diagram. **(C)** Major immune cell-types separated by lineage and shown as: 1) a zoomed-in region from one the six selected Voronoi diagrams, color-coded as in **(A, B)** (top panel), 2) a CODEX fluorescent image with Hoechst nuclear stain (blue) and the marker of interest (white) (middle panel), and 3) a simulated brightfield IHC image for the marker of interest (bottom panel). Scale bars: **(A, B)** 100 µm, **(C)** 10 µm.

The identification of immune cell-types has historically been based on the presence or absence of cluster of differentiation (CD) proteins, other cell surface markers, and lineage-specific transcription factors. Distinguishing cell-types in high dimensional tissue imaging data can be challenging due to 1) lateral marker spillover of cells adjacent to each other, 2) variable staining intensity of lineage-specific proteins across cell-types, and 3) simultaneously integration of lineage-specific marker expression patterns. Using higher-order unsupervised X-shift clustering (20) and manual refinement based on cell size and morphology, we were able to confidently stratify immune cell-types, even when they were bordering other lineage-specific immune cells (**Figure 3**). Two representative H&E-stained samples from the same tissue section, with six zoomed-in Voronoi diagrams representing the cell-types identified, are shown in **Figures 3A, B**. The major immune cell-types, stratified by lymphoid and myeloid lineages, are shown as Voronoi cell-type representations, 20x fluorescence images, and simulated brightfield images, which recreate the staining pattern that would be observed by conventional IHC using chromogenic methods (**Figure 3C**, top to bottom). Together, these data demonstrate the capability of this well-designed and titrated panel to effectively enumerate major immune cell-types and visualize their location within the overall TME architecture.

**Figure 4:**
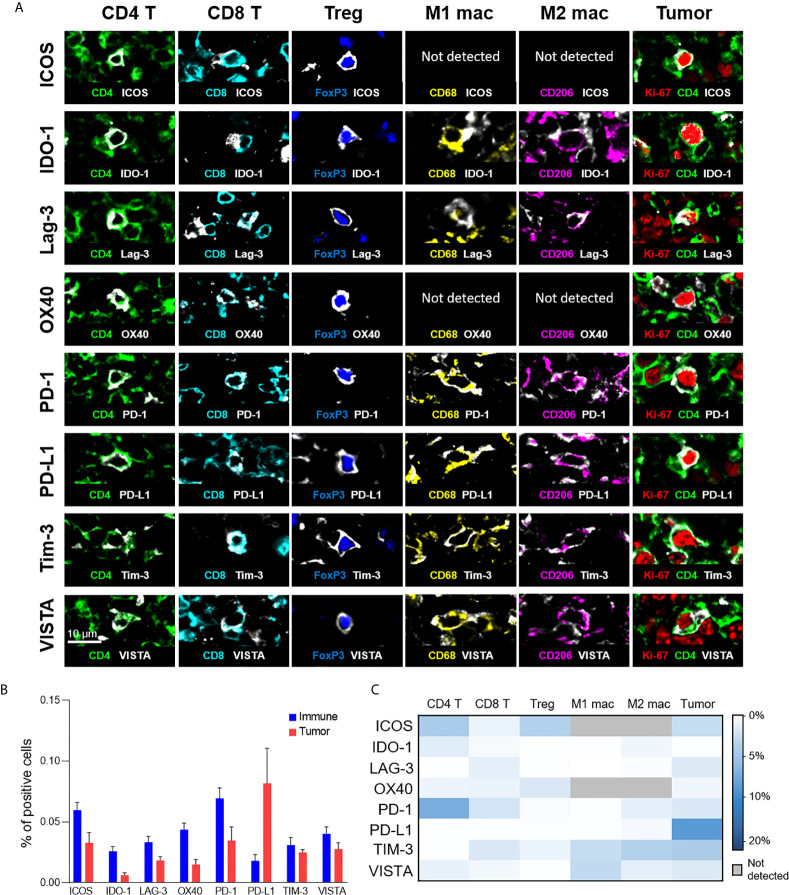
Expression of immunoregulatory proteins on different cell-types. **(A)** Color overlays of immune (CD4 – green; CD8 – cyan; FoxP3 – blue; CD68 – yellow; CD206 – magenta), tumor (Ki-67 – red; CD4 – green), and immunoregulatory proteins (ICOS, IDO-1, LAG-3, OX40, PD-1, PD-L1, TIM-3, VISTA – white). **(B)** Percentage of immune (T cells and macrophages) and tumor cells expressing immunoregulatory proteins; mean and standard error across patients. **(C)** Heatmap showing the mean percentage of immunoregulatory protein expression for the different T cell, macrophage, and tumor cell populations.

In addition to phenotypic stratification, we leveraged upon the high multiplexing capabilities of CODEX to include functional markers, particularly immunoregulatory proteins that are essential for the study of cancer immunology and immunotherapy. Specifically, we focused on eight functional immune molecules that are the targets of approved or in trial immunotherapies: ICOS, IDO-1, LAG-3, OX40, PD-1, PD-L1, TIM-3, and VISTA (**Figure 4A**) (2, 33–37). Representative examples of the staining pattern of these markers on T cells, macrophages, and tumor cells are shown (**Figure 4A**). Protein expression comparisons of immune and tumor cells reveal that PD-L1 was predominantly expressed on tumor cells, while ICOS, IDO-1, LAG-3, and OX40 were predominantly present on immune cells, and TIM-3 and VISTA were expressed on both tumor and immune cells in similar proportions (**Figure 4B**). The expression of each immunoregulatory protein varies across immune cell-types and individual patients (**Figure 4C** and **Supplementary Figure 6**), consistent with previous reports (26, 38–41). For example, PD-1 was strongly expressed on CD4^+^ T cells, ICOS on CD4^+^ T cells and Tregs, TIM-3 on M1 and M2 macrophages, and VISTA on M1 macrophages. These results demonstrate how simultaneous analysis of phenotypic and functional markers on single-cells, within their native spatial, tissue context, is a powerful approach for understanding the diverse landscape of functional immune molecules in cancer, and their roles in the immunotherapy responsiveness across patients.

## Discussion

Advancements in cancer immunotherapies require intimate knowledge of the orchestrated interaction and organization of cancer and immune cells. Multiplexed immunophenotyping approaches are essential in our efforts to identify predictive biomarkers of response and reveal insights into therapeutic mechanisms of action. Established, clinically accessible mIHC tissue imaging techniques are limited by the number of markers (≤ 7), while other multiparameter technologies (e.g., mass cytometry (CyTOF) and single-cell RNAseq) lack spatial context. The CODEX multiplexed tissue imaging method utilized in this study overcomes these limitations, and the 56-marker antibody panel established here enables extensive immunophenotyping of archival FFPE cancer specimens. Construction of a panel that captures structural markers, major immune cell-types and markers that inform cellular functional states (e.g., Ki-67 for proliferation and granzyme B for cytotoxicity), including immunoregulatory proteins, is paramount towards this goal. This work presents a blueprint for providing novel insights into the spatial organization and functional status of the TME, which are critical for advancing the field of cancer immunotherapy.

An important prerequisite for the study of FFPE tissues is the need for antigen retrieval, which involves the reversal of crosslinks formed during formalin fixation, to make epitopes accessible for antibody binding. Antigen retrieval involves boiling tissues in a specified pH buffer (ranging from pH 3 to 10) and detergents. Since the pH can greatly influence antibody binding (24, 42) and the entire CODEX antibody panel is stained simultaneously on a single tissue section, all antibodies must be optimized to perform with the same antigen retrieval protocol. The panel described here is optimized for antigen retrieval at pH 9, followed by antibody staining at 4°C overnight. In total, we conjugated 59 antibodies for implementation in this panel. 56 antibodies eventually passed our quality control assessment and were deemed as successfully conjugated and validated in FFPE human tissues, translating to a 94.9% success rate (i.e., 56/59 antibodies). The three antibodies that did not pass our assessment were TCR-α/β (clones G-11, T10B9), CTLA-4 (clones 2188A, BN13, L3D10), and TIGIT (clone TG1), likely owing to alteration of the antibody structure during the partial reduction step of the CODEX conjugation, the high pH antigen retrieval condition, aberrant oligonucleotide-antigen interactions, and/or low signal intensity.

Establishment a CODEX antibody panel, as described here, involves several considerations. First, antibodies must perform as expected when they intermixed with other antibodies. When combined, some antibodies may generate unexpected staining patterns not observed individually, due to aberrant cross-reactivity between antigens and/or oligonucleotides (43, 44). Second, multiple overlapping markers must be used to accurately classify cell-types. For instance, in this panel, Tregs were defined by co-expression of FoxP3, CD25, CD3, CD4, and CD45 (and lack of co-expression of non-lineage specific markers). This ability to visually inspect the spatial expression pattern of antibodies and cross-validate them against lineage-specific markers is a unique capability of highly multiplexed tissue imaging and an important milestone of antibody validation. Third, the multicycle panel order must be carefully considered. Importantly, after 10 cycles of iterative washing, hybridization, and stripping, we observe a slight decrease in signal strength of nuclear markers (16). Thus, antibodies that target nuclear markers and other low abundance proteins were placed in earlier cycles. As marker intensity and tissue morphology is not otherwise degraded during a multicycle (16, 18), the other markers were distributed to balance the panel across the fluorescent channels (i.e., Alexa488, ATTO550, and Alexa647). Fourth, to prevent fluorescent channel bleed through that could obscure a weaker signal, care must be taken to avoid combining within one cycle antibodies that stain different epitopes on the same cells at drastically different levels of intensity. For example, in this panel the helper T cell markers CD4 (strong staining) and CD5 (weak staining) were placed in different cycles. Finally, markers with low abundance, weak signal, and/or high background—generally this includes immunoregulatory proteins—should be imaged with the Alexa647 channel to overcome low signal-to-noise ratios, often from high tissue autofluorescence. As signal amplification is currently absent in CODEX, when further enhancement of the signal intensity is needed—herein for LAG-3—a reporter oligonucleotide with fluorescent tags on both the 3’ and 5’ ends should be used. A benefit of CODEX antibody panels is that they are customizable, allowing new tumor, immune, signaling, and drug target markers to be added as needed. Additionally, antibodies included in previously described CODEX panels (16, 18, 19) are compatible with the current panel and can be incorporated in the future as more unique oligonucleotides are validated and disclosed.

Immunotherapy is achieved through disruption of specific cell-to-cell interactions, resulting in activation of tumor surveillance by the native immune system. While such treatments are increasingly first-line for numerous cancers, not all patients derive benefit. The success or failure of immunotherapy likely depends on the balance between the expression of the drug-targeted immunoregulatory protein on immune and tumor cells as well as their location within the TME (19, 45–47). Multiplexed imaging studies utilizing 20-60 markers are generally required to achieve novel biomarker discovery studies (16, 19, 23, 26, 48). The 56-marker panel described here enables rigorous immunophenotyping and incorporates eight high value immunomodulatory proteins—ICOS, IDO-1, LAG-3, OX40, PD-1, PD-L1, TIM-3, and VISTA—that will empower further work into a better mechanistic understanding of immunotherapeutic responses (2, 33–37). This study also identified major differences in immunoregulatory protein expression between cell-types in CTCL, consistent with that previously observed with other tumor types (40). For example, PD-1 and ICOS were strongly expressed on CD4^+^ T cells, whereas TIM-3 and VISTA were strongly expressed on macrophages. Furthermore, immunoregulatory protein expression was highly variable across CTCL patients, in line with previous reports (40, 49); some patients had high expression of numerous markers whereas others had low expression of all markers. This degree of heterogeneity highlights the importance of analyzing functional immune molecules in individual patients and larger patient cohorts. Additionally, since the tissue microarray cores were obtained from only one area of the skin tumor biopsy, it is pertinent to incorporate several tissue microarray cores from the same sample or the whole tumor section to account for intra-tumor heterogeneity (50–53).

In summary, we developed, optimized, and applied a 56-marker antibody panel to analyze FFPE tumors by CODEX, exemplified by its application to CTCL. This panel allows the composition, location, and cellular state (e.g., proliferation or cytotoxicity) of TME components to be assessed relative to the expression of trial-targeted immunoregulatory proteins on immune and tumor cells. Since the CODEX antibody conjugation method is similar to that of other multiplexed imaging platforms, it is likely that the panel described here will be compatible with MIBI (25, 26), IMC (22), imaging cycler microscopy (ICM) (54), DNA exchange imaging (DEI) (55), MultiOmyx (MxIF) (56), and t-CyCIF (29). In our laboratory, we have had general success in antibody clone transfer between the CODEX and MIBI platforms. In fact, 22 of 56 antibodies included in this CODEX panel have been utilized in prior MIBI studies (26–28). While slight modifications to the antibody dilutions may be required when applying this panel across platforms (i.e., to account for small alterations in signal-to-noise ratios stemming from the mode of antibody detection: oligonucleotides in CODEX versus metals in MIBI and IMC), working knowledge of this panel will save considerable time, effort, and resources for researchers interested in studying the TME and immunotherapy responsiveness with multiplexed tissue imaging approaches. Ultimately, this panel allows for high-dimensional, spatially resolved characterization of the TME and offers unprecedented insights into tumor immunology, tissue architecture, the discovery of immunotherapy biomarkers, and potential applications beyond.

## Data Availability Statement

The raw data supporting the conclusions of this article will be made available by the authors, without reservation.

## Ethics Statement

The studies involving human participants were reviewed and approved by Stanford University IRB Administrative Panels on Human Subjects in Medical Research (HSR 46894). Since fully de-identified, this work was not considered human subjects research. The patients/participants provided their written informed consent to participate in this study.

## Author Contributions

DP initiated the study, conjugated antibodies, performed experiments, created figures and tables, and wrote the manuscript. CMS validated antibody stainings and revised the manuscript. MSK and YHK obtained clinical samples, provided clinical data, and revised the manuscript. GPN supervised the study and revised the manuscript. SJ initiated/supervised the study, performed computational analysis, created figures, and wrote the manuscript. All authors contributed to the article and approved the submitted version.

## Funding

This work was supported by the National Institutes of Health (NIH) 2U19AI057229-16 (GN), 5P01HL10879707 (GN), 5R01GM10983604 (GN), 5R33CA18365403 (GN), 5U01AI101984-07 (GN), 5UH2AR06767604 (GN), 5R01CA19665703 (GN), 5U54CA20997103 (GN), 5F99CA212231-02 (GN), 1F32CA233203-01 (GN), 5U01AI140498-02 (GN), 1U54HG010426-01 (GN), 5U19AI100627-07 (GN), 1R01HL120724-01A1 (GN), R33CA183692 (GN), R01HL128173-04 (GN), 5P01AI131374-02 (GN), 5UG3DK114937-02 (GN), 1U19AI135976-01 (GN), IDIQ17X149 (GN), 1U2CCA233238-01 (GN), 1U2CCA233195-01 (GN), F32CA233203 (DP), T32AR007422 (DP), The Bill and Melinda Gates Foundation OPP1113682 (GN) and INV-002704 (SJ and GN), The Cancer Research Institute (GN), The Parker Institute for Cancer Immunotherapy (GN), The Rachford & Carlotta A. Harris Endowed Chair (GN), and The Beckman Center for Molecular and Genetic Medicine (DP, CS, YK, and GN). DP was also supported by a Stanford Dean’s Fellowship and a Stanford Cancer Institute Fellowship. CS was supported by an Advanced Postdoc Mobility Fellowship from the Swiss National Science Foundation (P300PB_171189 and P400PM_183915) and an International Award for Research in Leukemia from the Lady Tata Memorial Trust, London, UK. SJ was supported by the Leukemia & Lymphoma Society Career Development Program and the Stanford Dean’s Fellowship.

## Conflict of Interest

GPN is a co-founder and stockholder of Akoya Biosciences, Inc. and an inventor on patent US9909167. CMS is a scientific advisor to Enable Medicine, LLC.

The remaining authors declare that the research was conducted in the absence of any commercial or financial relationships that could be construed as a potential conflict of interest.

## Supplementary Material

Supplementary Figure 1 Single-staining validations for the major immune cell-types around a germinal center in tonsil tissue. Four color images of APCs (specifically DCs), B cells, CD4^+^ T cells, CD8^+^ T cells, granulocytes, M1 macrophages, M2 macrophages, mast cells, NK cells, plasma cells, and Tregs are shown, with the key marker for the cell-type of interest (cyan), an positive control (red), negative control (green), and DRAQ5 nuclear marker (blue). Scale bars, 100 µm.

Supplementary Figure 2 Experimental workflow for CODEX. Clinical tissue specimens are stained using the 56-marker panel of antibodies conjugated to unique DNA oligonucleotides. A multicycle experiment is performed, with iterative cycles of hybridization of corresponding fluorescent oligonucleotides, imaging, and chemical stripping of fluorescent oligonucleotides. This results in a 58-dimensional image depicting protein expression for the 56 antibodies and 2 nuclear markers (Hoechst and DRAQ5), which can be used to generate 7-color overlay images and for subsequent analysis.

Supplementary Figure 3 Tissue microarray of eight CTCL tumor samples. **(A)** H&E staining, **(B)** Hoechst (nuclear) staining, and **(C)** seven-color overlay images of cytokeratin (green), vimentin (blue) CD31 (gray), Ki-67 (red), CD45 (cyan), CD3 (magenta), and CD68 (yellow). Scale bars, 100 µm.

Supplementary Figure 4 CTCL tissue stained with a 56-marker CODEX antibody panel. A single tissue region with epithelium (top left in each image) and dermis (remainder of image) is depicted in false gray color for each antibody. H&E and Hoechst (nuclear) stainings are also shown. Scale bar, 100 µm.

Supplementary Figure 5 Quantification of marker expression level. The distribution of expression is plotted for each of the 56 antibodies and DRAQ5 per patient sample. The black dot represents the median expression and the black lines represent the standard deviation.

Supplementary Figure 6 Expression of immunoregulatory proteins in T cells, macrophages, and tumor cells. **(A–E)** Percentage of marker-positive **(A)** CD4 T cells, **(B)** CD8 T cells, **(C)** Tregs, **(D)** M1 macrophages, **(E)** M2 macrophages as a percentage of all immune cells. **(F)** Percentage of marker-positive tumor cells as a percentage of all tumor cells. Marker expression is shown individually for the 8 CTCL patients. Gray boxes indicate that the marker is not detected in that cell-type.

## References

[1] Darvin P Toor SM Sasidharan Nair V Elkord E. Immune Checkpoint Inhibitors: Recent Progress and Potential Biomarkers. Exp Mol Med (2018) 50(12):1–11. 10.1038/s12276-018-0191-1 PMC6292890 30546008

[2] Sharma P Allison JP. The Future of Immune Checkpoint Therapy. Science (2015) 348(6230):56–61. 10.1126/science.aaa8172 25838373

[3] Xin Yu J Hubbard-Lucey VM Tang J. Immuno-Oncology Drug Development Goes Global. Nat Rev Drug Discov (2019) 18(12):899–900. 10.1038/d41573-019-00167-9 31780841

[4] Marin-Acevedo JA Dholaria B Soyano AE Knutson KL Chumsri S Lou Y. Next Generation of Immune Checkpoint Therapy in Cancer: New Developments and Challenges. J Hematol Oncol (2018) 11(1):39. 10.1186/s13045-018-0582-8 29544515 PMC5856308

[5] Liu M Wang X Wang L Ma X Gong Z Zhang S. Targeting the IDO1 Pathway in Cancer: From Bench to Bedside. J Hematol Oncol (2018) 11(1):100. 10.1186/s13045-018-0644-y 30068361 PMC6090955

[6] Qin S Xu L Yi M Yu S Wu K Luo S. Novel Immune Checkpoint Targets: Moving Beyond PD-1 and CTLA-4. Mol Cancer (2019) 18(1):155. 10.1186/s12943-019-1091-2 31690319 PMC6833286

[7] Mazzarella L Morganti S Marra A Trapani D Tini G Pelicci P. Master Protocols in Immuno-Oncology: do Novel Drugs Deserve Novel Designs? J Immunother Cancer (2020) 8(1):e000475. 10.1136/jitc-2019-000475 32238471 PMC7174064

[8] Aspeslagh S Postel-Vinay S Rusakiewicz S Soria JC Zitvogel L Marabelle A. Rationale for anti-OX40 Cancer Immunotherapy. Eur J Cancer (2016) 52:50–66. 10.1016/j.ejca.2015.08.021 26645943

[9] Schmidt C. The Benefits of Immunotherapy Combinations. Nature (2017) 552(7685):S67–S9. 10.1038/d41586-017-08702-7 29293245

[10] Swart M Verbrugge I Beltman JB. Combination Approaches With Immune-Checkpoint Blockade in Cancer Therapy. Front Oncol (2016) 6:233. 10.3389/fonc.2016.00233 27847783 PMC5088186

[11] Yu C Liu X Yang J Zhang M Jin H Ma X. Combination of Immunotherapy With Targeted Therapy: Theory and Practice in Metastatic Melanoma. Front Immunol (2019) 10:990. 10.3389/fimmu.2019.00990 31134073 PMC6513976

[12] Patel SA Minn AJ. Combination Cancer Therapy With Immune Checkpoint Blockade: Mechanisms and Strategies. Immunity (2018) 48(3):417–33. 10.1016/j.immuni.2018.03.007 PMC6948191 29562193

[13] Schmidt EV Chisamore MJ Chaney MF Maradeo ME Anderson J Baltus GA. Assessment of Clinical Activity of PD-1 Checkpoint Inhibitor Combination Therapies Reported in Clinical Trials. JAMA Netw Open (2020) 3(2):e1920833. 10.1001/jamanetworkopen.2019.20833 32049290 PMC12549068

[14] Havel JJ Chowell D Chan TA. The Evolving Landscape of Biomarkers for Checkpoint Inhibitor Immunotherapy. Nat Rev Cancer (2019) 19(3):133–50. 10.1038/s41568-019-0116-x PMC6705396 30755690

[15] Lu S Stein JE Rimm DL Wang DW Bell JM Johnson DB. Comparison of Biomarker Modalities for Predicting Response to PD-1/PD-L1 Checkpoint Blockade: A Systematic Review and Meta-Analysis. JAMA Oncol (2019) 5:1195–204. 10.1001/jamaoncol.2019.1549 PMC6646995 31318407

[16] Schurch CM Bhate SS Barlow GL Phillips DJ Noti L Zlobec I. Coordinated Cellular Neighborhoods Orchestrate Antitumoral Immunity at the Colorectal Cancer Invasive Front. Cell (2020) 182(5):1341–59.e19. 10.1016/j.cell.2020.07.005 32763154 PMC7479520

[17] Goltsev Y Samusik N Kennedy-Darling J Bhate S Hale M Vazquez G. Deep Profiling of Mouse Splenic Architecture With CODEX Multiplexed Imaging. Cell (2018) 174(4):968–81.e15. 10.1016/j.cell.2018.07.010 30078711 PMC6086938

[18] Black S Philliips D Hickey JW Kennedy-Darling J Venkataraaman VG Samusik N. CODEX Multiplexed Tissue Imaging With DNA-Conjugated Antibodies. Nat Protoc (2021). 10.1038/s41596-021-00556-8 PMC8647621 34215862

[19] Phillips D Matusiak M Gutierrez BR Bhate SS Barlow GL Jiang S. Immune Cell Topography Predicts Response to PD-1 Blockade in Cutaneous T Cell Lymphoma. medRxiv (2020). 10.1101/2020.12.06.20244913 PMC8602403 34795254

[20] Samusik N Good Z Spitzer MH Davis KL Nolan GP. Automated Mapping of Phenotype Space With Single-Cell Data. Nat Methods (2016) 13(6):493–6. 10.1038/nmeth.3863 PMC4896314 27183440

[21] Kennedy-Darling J Bhate SS Hickey JW Black S Barlow GL Vazquez G. Highly Multiplexed Tissue Imaging Using Repeated Oligonucleotide Exchange Reaction. Eur J Immunol (2021) 51:1262–77. 10.1002/eji.202048891 PMC8251877 33548142

[22] Giesen C Wang HA Schapiro D Zivanovic N Jacobs A Hattendorf B. Highly Multiplexed Imaging of Tumor Tissues With Subcellular Resolution by Mass Cytometry. Nat Methods (2014) 11(4):417–22. 10.1038/nmeth.2869 24584193

[23] Jackson HW Fischer JR Zanotelli VRT Ali HR Mechera R Soysal SD. The Single-Cell Pathology Landscape of Breast Cancer. Nature (2020) 578(7796):615–20. 10.1038/s41586-019-1876-x 31959985

[24] Ijsselsteijn ME van der Breggen R Farina Sarasqueta A Koning F de Miranda N. A 40-Marker Panel for High Dimensional Characterization of Cancer Immune Microenvironments by Imaging Mass Cytometry. Front Immunol (2019) 10:2534. 10.3389/fimmu.2019.02534 31736961 PMC6830340

[25] Angelo M Bendall SC Finck R Hale MB Hitzman C Borowsky AD. Multiplexed Ion Beam Imaging of Human Breast Tumors. Nat Med (2014) 20(4):436–42. 10.1038/nm.3488 PMC4110905 24584119

[26] Keren L Bosse M Marquez D Angoshtari R Jain S Varma S. A Structured Tumor-Immune Microenvironment in Triple Negative Breast Cancer Revealed by Multiplexed Ion Beam Imaging. Cell (2018) 174(6):1373–87.e19. 10.1016/j.cell.2018.08.039 30193111 PMC6132072

[27] Keren L Bosse M Thompson S Risom T Vijayaragavan K McCaffrey E. MIBI-TOF: A Multiplexed Imaging Platform Relates Cellular Phenotypes and Tissue Structure. Sci Adv (2019) 5(10):eaax5851. 10.1126/sciadv.aax5851 31633026 PMC6785247

[28] Ji AL Rubin AJ Thrane K Jiang S Reynolds DL Meyers RM. Multimodal Analysis of Composition and Spatial Architecture in Human Squamous Cell Carcinoma. Cell (2020) 182(6):1661–2. 10.1016/j.cell.2020.08.043 PMC7505493 32946785

[29] Lin JR Izar B Wang S Yapp C Mei S Shah PM. Highly Multiplexed Immunofluorescence Imaging of Human Tissues and Tumors Using t-CyCIF and Conventional Optical Microscopes. Elife (2018) 7:e31657. 10.7554/eLife.31657 29993362 PMC6075866

[30] Goldblum JR Lamps LW McKenney JK Myers JL Ackerman LV Rosai J. Rosai and Ackerman’s Surgical Pathology. Eleventh edition Vol. 2 volumes. Philadelphia, PA: Elsevier (2018). xiv, 2142 pages.

[31] Uhlen M Oksvold P Fagerberg L Lundberg E Jonasson K Forsberg M. Towards a Knowledge-Based Human Protein Atlas. Nat Biotechnol (2010) 28(12):1248–50. 10.1038/nbt1210-1248 21139605

[32] Uhlen M Bandrowski A Carr S Edwards A Ellenberg J Lundberg E. A Proposal for Validation of Antibodies. Nat Methods (2016) 13(10):823–7. 10.1038/nmeth.3995 PMC10335836 27595404

[33] Andrews LP Marciscano AE Drake CG Vignali DA. LAG3 (CD223) as a Cancer Immunotherapy Target. Immunol Rev (2017) 276(1):80–96. 10.1111/imr.12519 28258692 PMC5338468

[34] Kwa MJ Adams S. Checkpoint Inhibitors in Triple-Negative Breast Cancer (TNBC): Where to Go From Here. Cancer (2018) 124(10):2086–103. 10.1002/cncr.31272 29424936

[35] Marshall HT Djamgoz MBA. Immuno-Oncology: Emerging Targets and Combination Therapies. Front Oncol (2018) 8:315. 10.3389/fonc.2018.00315 30191140 PMC6115503

[36] Murciano-Goroff YR Warner AB Wolchok JD. The Future of Cancer Immunotherapy: Microenvironment-Targeting Combinations. Cell Res (2020) 30(6):507–19. 10.1038/s41422-020-0337-2 PMC7264181 32467593

[37] Liebler DC Holzer TR Haragan A Morrison RD O’Neill Reising L Ackermann BL. Analysis of Immune Checkpoint Drug Targets and Tumor Proteotypes in Non-Small Cell Lung Cancer. Sci Rep (2020) 10(1):9805. 10.1038/s41598-020-66902-0 32555523 PMC7300007

[38] Herbst RS Soria JC Kowanetz M Fine GD Hamid O Gordon MS. Predictive Correlates of Response to the Anti-PD-L1 Antibody MPDL3280A in Cancer Patients. Nature (2014) 515(7528):563–7. 10.1038/nature14011 PMC4836193 25428504

[39] Tu L Guan R Yang H Zhou Y Hong W Ma L. Assessment of the Expression of the Immune Checkpoint Molecules PD-1, CTLA4, TIM-3 and LAG-3 Across Different Cancers in Relation to Treatment Response, Tumor-Infiltrating Immune Cells and Survival. Int J Cancer (2020) 147(2):423–39. 10.1002/ijc.32785 31721169

[40] Gorris MAJ Halilovic A Rabold K van Duffelen A Wickramasinghe IN Verweij D. Eight-Color Multiplex Immunohistochemistry for Simultaneous Detection of Multiple Immune Checkpoint Molecules Within the Tumor Microenvironment. J Immunol (2018) 200(1):347–54. 10.4049/jimmunol.1701262 29141863

[41] Li X Wang R Fan P Yao X Qin L Peng Y. A Comprehensive Analysis of Key Immune Checkpoint Receptors on Tumor-Infiltrating T Cells From Multiple Types of Cancer. Front Oncol (2019) 9:1066. 10.3389/fonc.2019.01066 31709176 PMC6823747

[42] Shi SR Shi Y Taylor CR. Antigen Retrieval Immunohistochemistry: Review and Future Prospects in Research and Diagnosis Over Two Decades. J Histochem Cytochem (2011) 59(1):13–32. 10.1369/jhc.2010.957191 21339172 PMC3201121

[43] Ellington AA Kullo IJ Bailey KR Klee GG. Antibody-Based Protein Multiplex Platforms: Technical and Operational Challenges. Clin Chem (2010) 56(2):186–93. 10.1373/clinchem.2009.127514 PMC2901849 19959625

[44] Wang W Lilyestrom WG Hu ZY Scherer TM. Cluster Size and Quinary Structure Determine the Rheological Effects of Antibody Self-Association At High Concentrations. J Phys Chem B (2018) 122(7):2138–54. 10.1021/acs.jpcb.7b10728 29359938

[45] Blank CU Haanen JB Ribas A Schumacher TN. Cancer Immunology. The “Cancer Immunogram”. Science (2016) 352(6286):658–60. 10.1126/science.aaf2834 27151852

[46] Tumeh PC Harview CL Yearley JH Shintaku IP Taylor EJ Robert L. PD-1 Blockade Induces Responses by Inhibiting Adaptive Immune Resistance. Nature (2014) 515(7528):568–71. 10.1038/nature13954 PMC4246418 25428505

[47] Gnjatic S Bronte V Brunet LR Butler MO Disis ML Galon J. Identifying Baseline Immune-Related Biomarkers to Predict Clinical Outcome of Immunotherapy. J Immunother Cancer (2017) 5:44. 10.1186/s40425-017-0243-4 28515944 PMC5432988

[48] Chevrier S Levine JH Zanotelli VRT Silina K Schulz D Bacac M. An Immune Atlas of Clear Cell Renal Cell Carcinoma. Cell (2017) 169(4):736–49.e18. 10.1016/j.cell.2017.04.016 28475899 PMC5422211

[49] Wang JB Li P Liu XL Zheng QL Ma YB Zhao YJ. An Immune Checkpoint Score System for Prognostic Evaluation and Adjuvant Chemotherapy Selection in Gastric Cancer. Nat Commun (2020) 11(1):6352. 10.1038/s41467-020-20260-7 33311518 PMC7732987

[50] Boddupalli CS Bar N Kadaveru K Krauthammer M Pornputtapong N Mai Z. Interlesional Diversity of T Cell Receptors in Melanoma With Immune Checkpoints Enriched in Tissue-Resident Memory T Cells. JCI Insight (2016) 1(21):e88955. 10.1172/jci.insight.88955 28018970 PMC5161225

[51] Madore J Vilain RE Menzies AM Kakavand H Wilmott JS Hyman J. PD-L1 Expression in Melanoma Shows Marked Heterogeneity Within and Between Patients: Implications for Anti-PD-1/PD-L1 Clinical Trials. Pigment Cell Melanoma Res (2015) 28(3):245–53. 10.1111/pcmr.12340 25477049

[52] Mansfield AS Murphy SJ Peikert T Yi ES Vasmatzis G Wigle DA. Heterogeneity of Programmed Cell Death Ligand 1 Expression in Multifocal Lung Cancer. Clin Cancer Res (2016) 22(9):2177–82. 10.1158/1078-0432.CCR-15-2246 PMC4854782 26667490

[53] Wei Y Zhao Q Gao Z Lao XM Lin WM Chen DP. The Local Immune Landscape Determines Tumor PD-L1 Heterogeneity and Sensitivity to Therapy. J Clin Invest (2019) 129(8):3347–60. 10.1172/JCI127726 PMC6668685 31112529

[54] Schubert W Bonnekoh B Pommer AJ Philipsen L Bockelmann R Malykh Y. Analyzing Proteome Topology and Function by Automated Multidimensional Fluorescence Microscopy. Nat Biotechnol (2006) 24(10):1270–8. 10.1038/nbt1250 17013374

[55] Wang Y Woehrstein JB Donoghue N Dai M Avendano MS Schackmann RCJ. Rapid Sequential in Situ Multiplexing With DNA Exchange Imaging in Neuronal Cells and Tissues. Nano Lett (2017) 17(10):6131–9. 10.1021/acs.nanolett.7b02716 PMC5658129 28933153

[56] Gerdes MJ Sevinsky CJ Sood A Adak S Bello MO Bordwell A. Highly Multiplexed Single-Cell Analysis of Formalin-Fixed, Paraffin-Embedded Cancer Tissue. Proc Natl Acad Sci U S A (2013) 110(29):11982–7. 10.1073/pnas.1300136110 PMC3718135 23818604

