# Supplementary figures and images for: Highly Multiplexed Phenotyping of Immunoregulatory Proteins in the Tumor Microenvironment by CODEX Tissue Imaging
Source: Front Immunol. 2021 May 19;12:687673. doi: 10.3389/fimmu.2021.687673 (PMC8170307; doi:10.3389/fimmu.2021.687673)

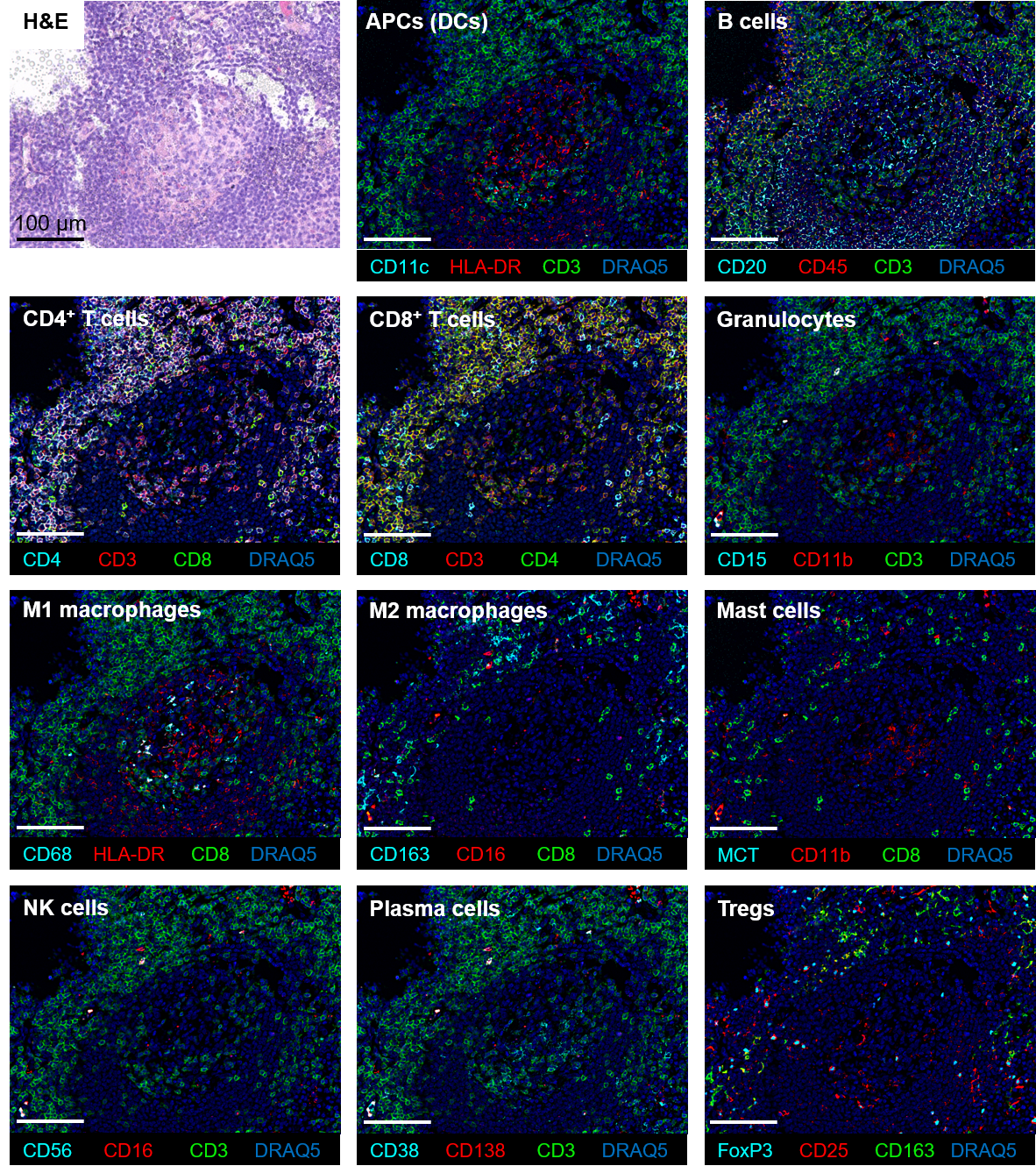

Supplement: Supplementary Figure 1 — Single-staining validations for the major immune cell-types around a germinal center in tonsil tissue. Four color images of APCs (specifically DCs), B cells, CD4+ T cells, CD8+ T cells, granulocytes, M1 macrophages, M2 macrophages, mast cells, NK cells, plasma cells, and Tregs are shown, with the key marker for the cell-type of interest (cyan), an positive control (red), negative control (green), and DRAQ5 nuclear marker (blue). Scale bars, 100 µm. [file Image_1.tif]

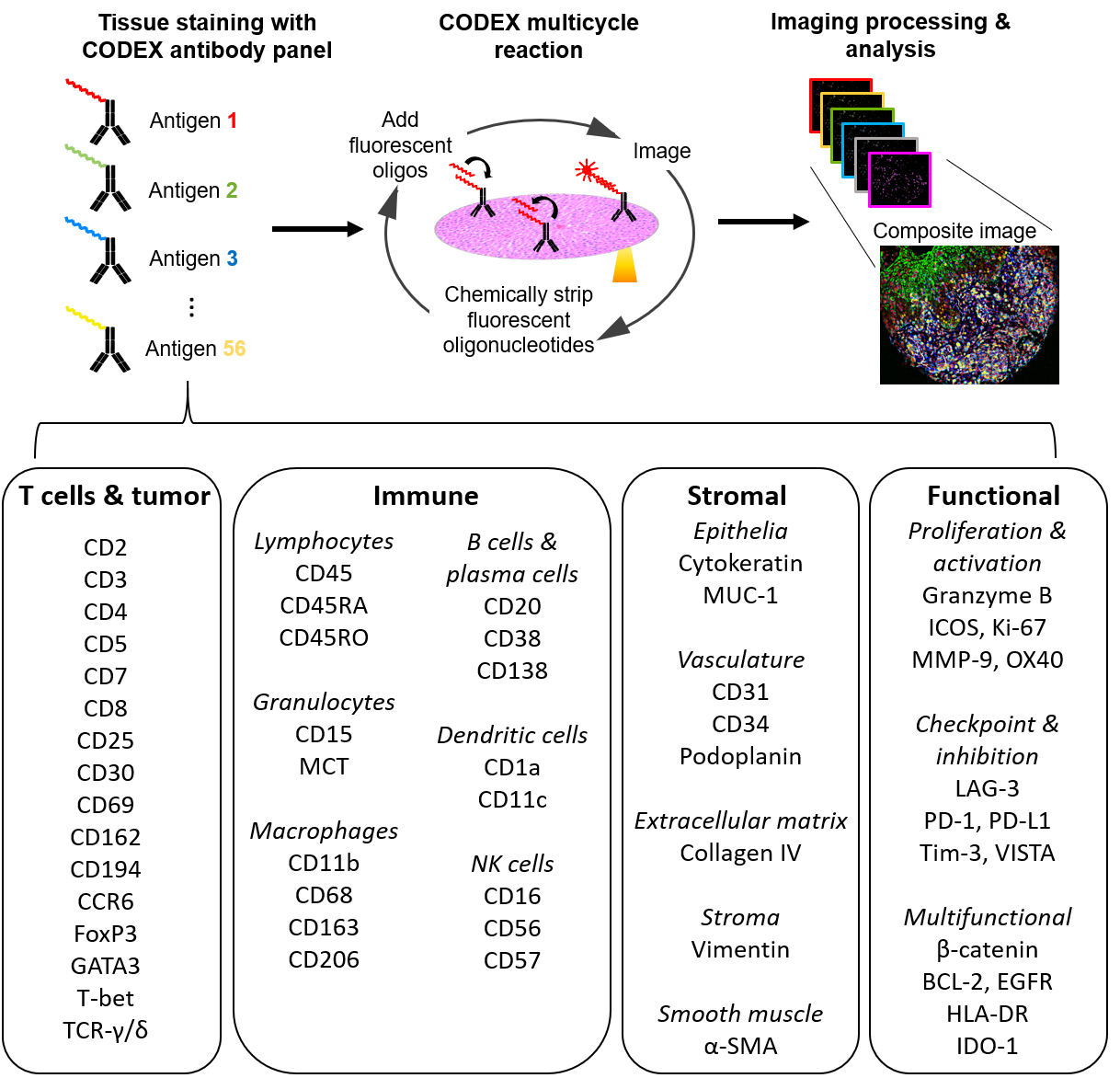

Supplement: Supplementary Figure 2 — Experimental workflow for CODEX. Clinical tissue specimens are stained using the 56-marker panel of antibodies conjugated to unique DNA oligonucleotides. A multicycle experiment is performed, with iterative cycles of hybridization of corresponding fluorescent oligonucleotides, imaging, and chemical stripping of fluorescent oligonucleotides. This results in a 58-dimensional image depicting protein expression for the 56 antibodies and 2 nuclear markers (Hoechst and DRAQ5), which can be used to generate 7-color overlay images and for subsequent analysis. [file Image_2.tif]

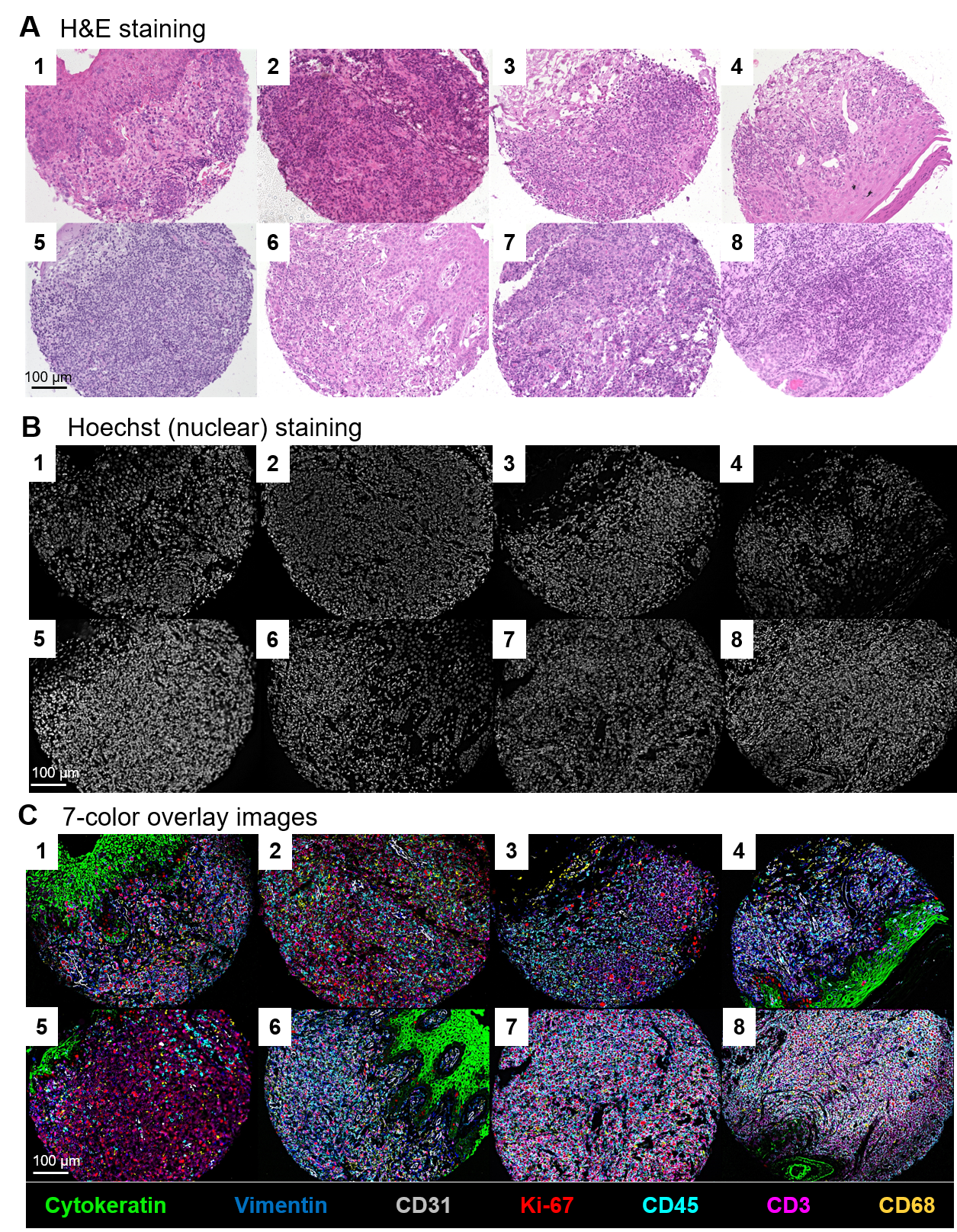

Supplement: Supplementary Figure 3 — Tissue microarray of eight CTCL tumor samples. (A) H&E staining, (B) Hoechst (nuclear) staining, and (C) seven-color overlay images of cytokeratin (green), vimentin (blue) CD31 (gray), Ki-67 (red), CD45 (cyan), CD3 (magenta), and CD68 (yellow). Scale bars, 100 µm. [file Image_3.tif]

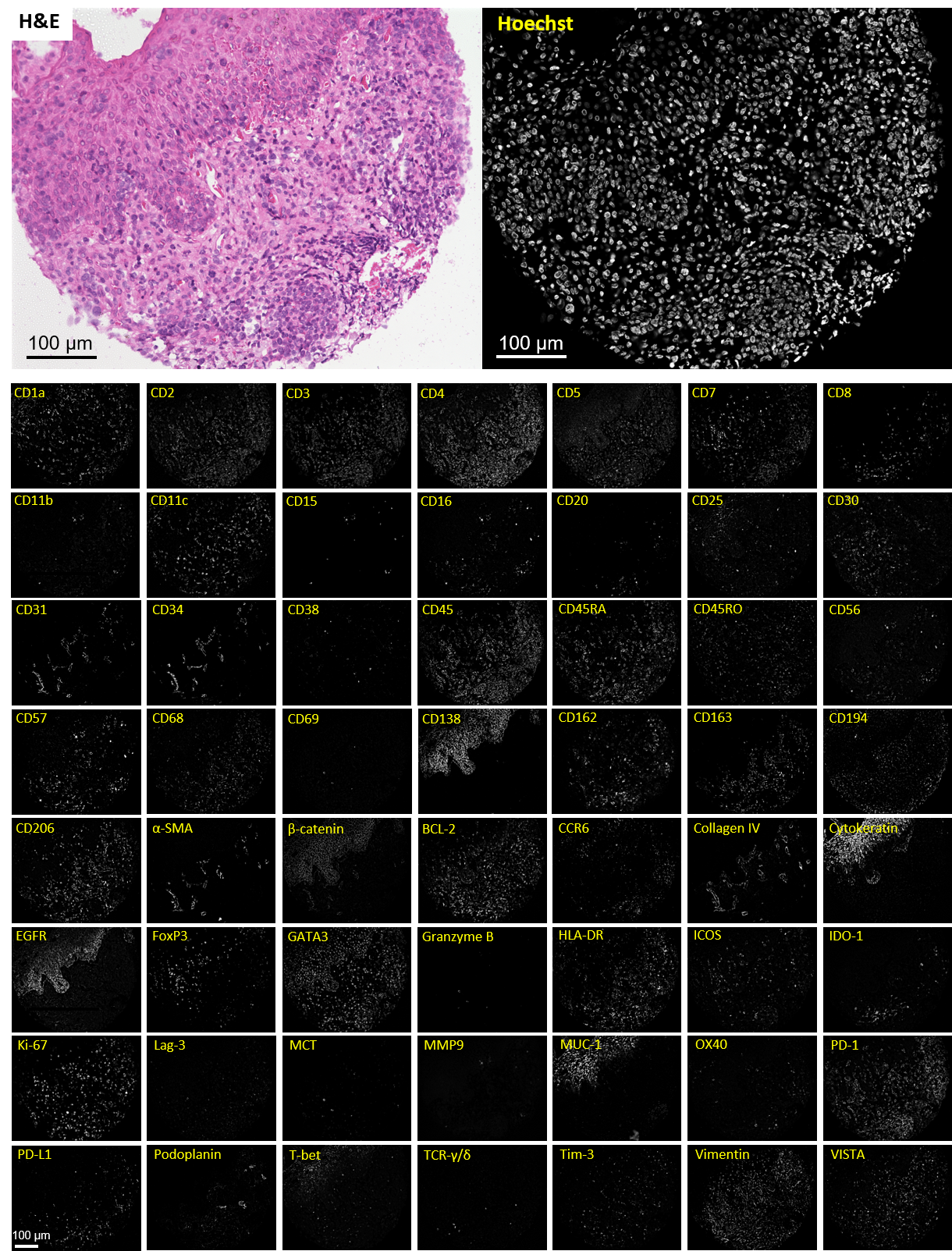

Supplement: Supplementary Figure 4 — CTCL tissue stained with a 56-marker CODEX antibody panel. A single tissue region with epithelium (top left in each image) and dermis (remainder of image) is depicted in false gray color for each antibody. H&E and Hoechst (nuclear) stainings are also shown. Scale bar, 100 µm. [file Image_4.tif]

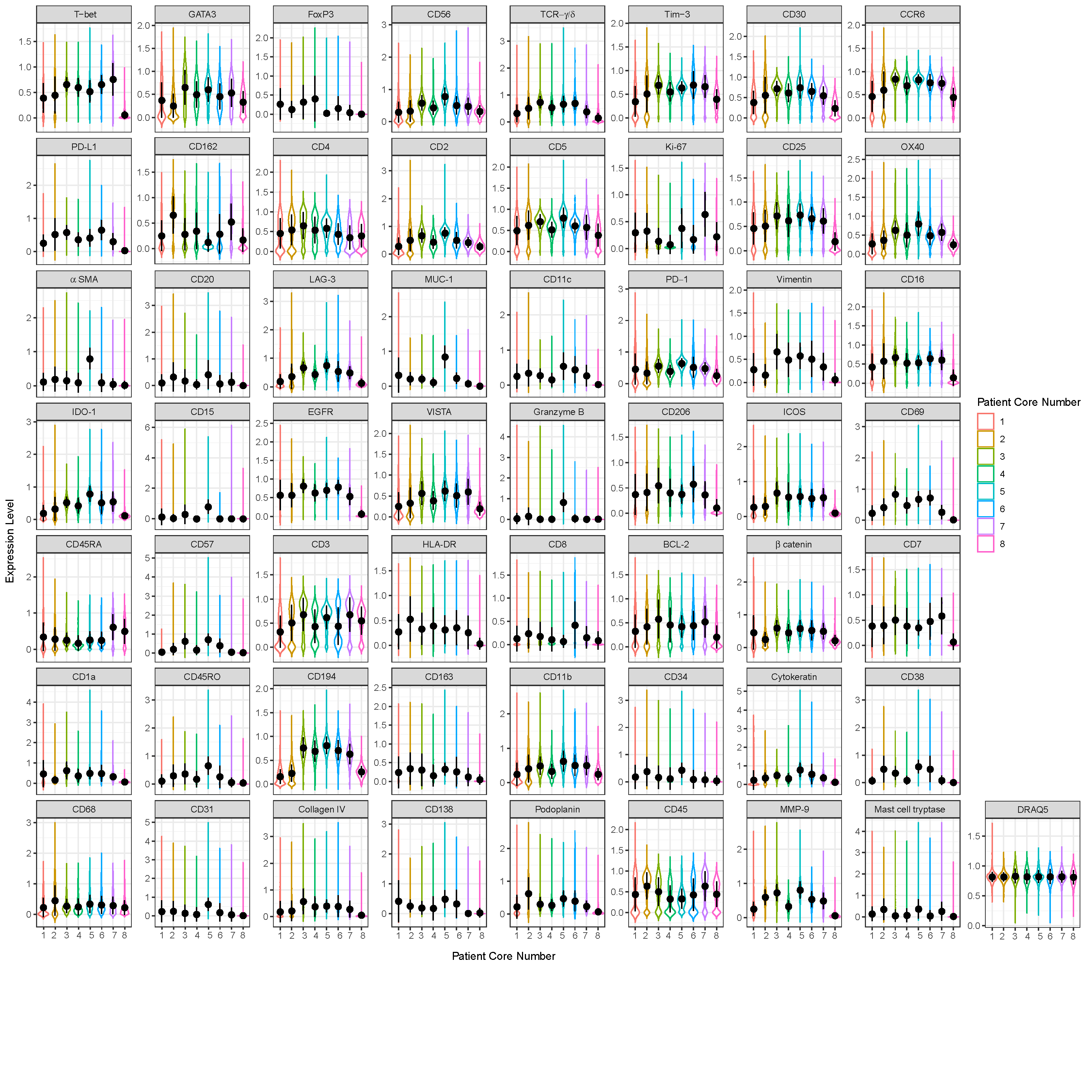

Supplement: Supplementary Figure 5 — Quantification of marker expression level. The distribution of expression is plotted for each of the 56 antibodies and DRAQ5 per patient sample. The black dot represents the median expression and the black lines represent the standard deviation. [file Image_5.tif]

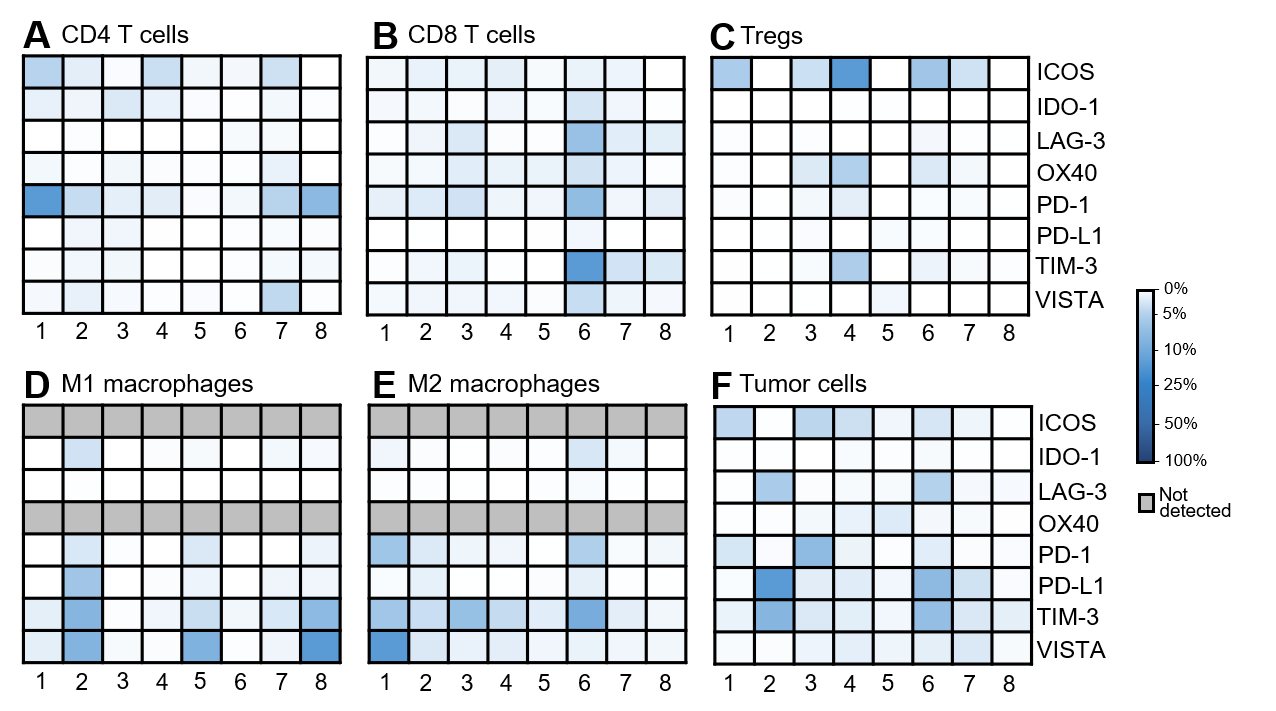

Supplement: Supplementary Figure 6 — Expression of immunoregulatory proteins in T cells, macrophages, and tumor cells. (A–E) Percentage of marker-positive (A) CD4 T cells, (B) CD8 T cells, (C) Tregs, (D) M1 macrophages, (E) M2 macrophages as a percentage of all immune cells. (F) Percentage of marker-positive tumor cells as a percentage of all tumor cells. Marker expression is shown individually for the 8 CTCL patients. Gray boxes indicate that the marker is not detected in that cell-type. [file Image_6.tif]
